# A Metabolomics Approach to Establish the Relationship between the Techno-Functional Properties and Metabolome of Indian Goat Yoghurt

**DOI:** 10.3390/foods13060913

**Published:** 2024-03-17

**Authors:** Hameedur Rehman, Kanchanpally Saipriya, Ashish Kumar Singh, Richa Singh, Ganga Sahay Meena, Yogesh Khetra, Heena Sharma

**Affiliations:** 1 Dairy Technology Division, ICAR-National Dairy Research Institute, Karnal 132001, Haryana, India; 2 Dairy Chemistry Division, ICAR-National Dairy Research Institute, Karnal 132001, Haryana, India

**Keywords:** yoghurt, goat milk, metabolites, regression, firmness, GC-MS

## Abstract

Introduction: Goat milk has poorer fermentation characteristics due to the absence or only traces of αs1-casein, due to which goat yoghurt contains a less dense gel structure. Moreover, the fermentation characteristics of the milk vary between the breeds of the same species. Therefore, it becomes imperative to explore a few metabolites which could regulate the techno-functional properties of goat yoghurt. Objectives: This study was aimed at relating the metabolite profile of yoghurt prepared from milk of *Barbari*, an indigenous goat breed of India, and its techno-functional properties (firmness, whey syneresis, and flow behaviour) using multivariate data analysis and regression models. Results: Goat yoghurt was prepared with two different total solids (TS) levels (12 and 16%) and cultures, namely, commercial culture comprising a thermophilic yoghurt culture (A) and NCDC-263 comprising a mixed yoghurt culture (B). Results demonstrated a significant difference (*p* < 0.05) in whey syneresis with the increase in the TS level. Flow behaviour of all yoghurt samples showed a decrease in viscosity with an increase in shear rate, which confirmed its non-Newtonian behaviour and shear thinning nature, whereas frequency sweep confirmed its viscoelastic nature. Firmness was the most affected under the influence of different TS and culture levels. It was higher (*p* < 0.05) for 16-A, followed by 16-3B, and minimum for 12-2B. GC-MS-based metabolomics of the yoghurt revealed a total of 102 metabolites, out of which 15 metabolites were differentially expressed (*p* < 0.05), including 2-hydroxyethyl palmitate, alpha-mannobiose, and myo-inositol. Multivariate data analysis revealed clear separation among groups using principal component analysis and several correlations using a correlation heat map. Further, regression analysis exhibited methylamine (0.669) and myo-inositol (0.947) with higher regression coefficients (R^2^ values) exceeding 0.6, thus demonstrating their significant influence on the techno-functional properties, mainly firmness, of the yogurt. Conclusion: In conclusion, A gas chromatography-based metabolomics approach could successfully establish a relationship between the metabolome and the techno-functional properties of the yoghurt.

## 1. Introduction

Yoghurt has been prepared since ancient times and is still consumed globally, with around 700 different varieties [1]. The unveiling of health benefits of yoghurt dates back to 1900s, when lactic acid bacteria used for the fermentation of milk was found to alter the intestinal microbiota for health-promoting activities [2]. Further, the industrial production of yoghurt commenced in 1919 by Danone company [3]. Earlier than this, the culture used for yoghurt production included *Lactobacillus bulgaricus* and *Streptococcus thermophilus.* Both the cultures were considered to work in a symbiotic relationship with each other until later it was found that other strains are also added to yoghurt for improving its flavour [4]. Leuconostoc is mainly used to produce a butter-like flavour in the yoghurt [5]. Owing to their health benefits, growing consumption has led to an increase in the demand for fermented milk and milk products. The typical manufacturing process of yoghurt involves standardization, homogenization, thermal treatment followed by fermentation, incubation and cooling of the product. The standardization of fat and non-fat solids is performed to achieve the desirable texture of the product and to meet any specific need of the consumer. Homogenization prevents the separation of fat, while the fermentation or inoculation of milk is performed with suitable culture at a desirable level in order to convert the lactose of milk to lactic acid, thereby bringing down the pH of the milk to 4.6. Two types of yoghurt are usually prepared following this method: stirred-type yoghurt, exhibiting a viscous and creamy texture, and set-type yoghurt, which displays a gel-like texture [6]. 

Recently, there has been a steady rise in demand for small ruminant milk, particularly goat milk, and the dairy market has seen an upsurge in goat milk products, as whole milk or fermented products [7]. Besides its nutritional advantages, goat milk possesses certain health virtues including hypoallergenicity and therapeutic properties due to its biologically active components, including peptides, medium-chain fatty acids, and oligosaccharides [8]. Although the majority of the dairy products available on the market globally are made of either cow milk and/or buffalo milk, better margins in goat milk products have attracted the attention of producers and investors in this field of dairy science [9]. Presently, several goat milk products are commercially available, including yoghurt, cheese, goat milk powder [10]. However, due to unique composition of goat milk as compared to cow milk (higher lactose-derived oligosaccharide content (0.25–0.3 g L^−1^) than cow milk (0.03–0.06 g L^−1^); higher percentage of medium-chain fatty acids (30–35%) than cow milk (15–20%)), there are certain technological challenges associated with the preparation of goat milk products [11]. The higher buffering capacity of goat milk is mainly due to the presence of phosphates, protein, and higher non-protein nitrogen than cow milk. These differences in the composition also result in different technological properties between the products prepared from cow and goat milk. Technological challenges such as weaker and less-dense gel, softer curd, lower curd tension, and a lower water holding capacity pose a hurdle for manufacturers to develop desirable and acceptable fermented goat milk products. All these factors result in the decreased acceptability of fermented goat milk products at a larger scale. Therefore, without suitable interventions, it becomes difficult for the manufacturers to prepare fermented goat milk product. Yoghurt is one such fermented goat milk product which needs technological interventions so that a thick, gel-like product is prepared by inoculation with starter culture under suitable conditions. Furthermore, there are several factors which can influence the quality of the end product, including the milk composition and the type of starter culture used. In addition, the total solid content of the milk affects the rheological and textural properties of the yoghurt [12]. Higher casein contents in milk have been related to the reinforcement of the protein matrix density that leads to an improved water holding capacity of the yoghurt gel.

The starter culture used in yoghurt is a combination of lactic acid bacteria (*Streptococcus thermophilus* and *Lactobacillus delbrueckii* subsp. *bulgaricus*), which produce exo-metabolites in their metabolism that are considered as key factors that determine the texture and aroma of the fermented products [13]. Yoghurt starter cultures exhibit an obligate symbiotic relationship during their growth in milk medium. Further, yoghurt can be considered as a complex food made up of hundreds of biomolecules, including proteins, lipids, carbohydrates, and various other small compounds such as amino acids, organic acids, nucleic acids, fatty acids, minerals, and aroma volatiles, which contribute to its unique techno-functional properties [14]. Metabolomics is a newly developed “omics” discipline, focused on the identification and quantification of small molecules (less than 1500 Da) within the metabolome [15]. Previous conventional techniques allow the analysis of macro-components of a food system; however, metabolomics not only provides a detailed nutritional profile of the food but also helps in monitoring the changes during the processing and storage of food at the molecular level [16]. The rapid identification and quantification of a specific component in a food provides the manufacturers the opportunity to assess food safety. Further, analysis of volatile components, which mainly contribute to the flavour of the food, provides opportunity for the production of food with desired flavour profiles [17]. Therefore, metabolomics has given impetus to the food industries for adulteration detection, the optimization of processing variables and ingredients, and the accuracy of food traceability. Furthermore, it mitigates the limitations of conventional techniques including a long time for identification and accuracy. Several techniques are used for the detection and identification of low-molecular-weight substances in a food system, including gas chromatography–mass spectrometry (GC-MS). GC-MS is considered as a powerful analytical tool for unravelling the metabolites generated during food processing, including fermented foods such as yoghurt. Several studies have employed metabolomics as an analytical tool for the detection, identification and quantification of low-molecular-weight substances in milk and milk products [18,19,20,21]. Milk fermented with various strains, including *Streptococcus thermophilus* S10, revealed 39 differentially regulated metabolites and suggested that thiol esters from short-chain fatty acids provide a unique flavour to fermented milk [22], while the metabolic profile of fermented milk with *Lactobacillus bulgaricus, Lacticaseibacillus paracasei* and *Kluyveromyces marxianus* exhibited 4535 metabolites during storage [23]. The key to all the research suggests that metabolites may vary according to different factors employed during the processing of milk for value addition. Among several other factors, factors which may affect the metabolite generation in yoghurt include the type and inoculation level of the starter culture, the incubation temperature, and the processing protocol [24]. In addition, the metabolite profile is greatly influenced by intrinsic factors such as the species and breed of the animal. To identify and quantify the various metabolites formed during milk fermentation, metabolomics techniques have been increasingly employed in recent years. Biochemical changes due to microbial activity during fermentation provides opportunities to predict the techno-functional and nutritional quality of fermented food products [25]. The role of traditional yoghurt starter culture to provide texture to yoghurt has been well documented; however, there are limited studies describing the metabolite generation during fermentation and their association with the resultant textural attributes of the yoghurt.

Considering all the above-mentioned data and the literature, it can be realized that the dairy industry has witnessed an increase in the demand for goat milk and milk products owing to their nutritional benefits. Further, the technological properties and functional attributes of goat milk are considered to be affected due to the presence of low-molecular-weight substances such as metabolites. In line with this, the present study includes the preparation of yoghurt from the milk of an Indian goat breed, namely, *Barbari,* and, further, analyses the metabolite profile and characterizes its techno-functional properties by varying the total solids concentration, and type and level of the starter culture. The present study could not only provide better understanding of the role of metabolites in yoghurt formation, as these metabolites are closely related to technological properties, but it also provided the scientific basis to establish the relationship between yoghurt metabolites and its techno-functional properties using a metabolomics approach.

## 2. Materials and Methods

### 2.1. Procurement of Goat Milk and Yoghurt Cultures

Fresh *Barbari* milk was procured immediately after milking from a herd of the *Barbari* breed maintained by the Livestock Research Centre, ICAR-National Dairy Research Institute, Karnal, Haryana, India. The collection of yoghurt cultures NCDC-263 was obtained from the National Collection of Dairy Cultures (NCDC), ICAR-National Dairy Research Institute, Karnal, Haryana, India. The culture ampoule was carefully propagated to prepare a mother culture followed by a bulk culture. An industrially procured commercial culture, YoFlex Express 1.0 from CHR HANSEN in Denmark, comprising a thermophilic yoghurt culture with a pack size of 50 U, was procured. The culture was blended with 500 mL of sterilized skim milk in a reagent bottle and incubated at 42 °C until it achieved the desired consistency. Once set, the yoghurt was gently stirred and utilized at a rate of 1 mL 1000 mL^−1^ of milk. All the chemicals utilized in the study were of analytical grade (AR) and were obtained from reputable suppliers. All reagents used in this study were freshly prepared following the standard procedures.

### 2.2. Processing and Preparation of Goat Milk Powder

Fresh *Barbari* goat milk was subjected to filtration using a muslin cloth to eliminate suspended particles, followed by pasteurization at 72 °C for 15 s. The milk was then concentrated to 24.3 g 100 mL^−1^ total solids using a steam jacketed kettle. Subsequently, the concentrated milk was subjected to spray drying at 170 °C, with a flow rate of 18–19 mL min^−1^. The resulting goat milk powder was then packaged in PET (polyester) containers and stored at a temperature of 25 °C.

### 2.3. Preparation of Goat Milk Yoghurt at Different Total Solids Contents Using Different Starter Culture

Goat milk yoghurt was prepared at two different total solids (TS) levels, i.e., 12% and 16%, by standardizing the milk using goat milk powder. The yoghurt mix was heat-treated at 90 ± 2 °C for 10 min in a water bath and then cooled to 42 °C. The cooled yoghurt mix was inoculated with required quantity of yoghurt culture, including commercial culture and NCDC-263, mixed well to ensure proper dispersion of culture, poured in a 150 mL beaker, fitted with lids (aluminium foil) and incubated at 42 °C until desirable titratable acidity (0.7% lactic acid and pH 4.6) was achieved. Yoghurt samples were then transferred to a refrigerator maintained at 4 ± 1 °C. The nomenclature of yoghurt samples prepared with different TS levels and culture levels is shown in Table 1. 

### 2.4. Analytical Methods

#### 2.4.1. Physico-Chemical Characteristics

The proximate composition of the yoghurt samples, i.e., total solids [26], the crude protein content [27], the fat content in skim milk [28] and yoghurt [29], and the ash content [30], was estimated using standard protocol. The pH of the samples was measured using a pH meter (PHAN LABINDIA Model, Labtek Engg. Pvt. Ltd., New Delhi, India) equipped with a combination electrode. The titratable acidity of the yoghurt was determined as a percentage of lactic acid using the established method described in IS: 1479 (Part I) [31]. The colour values (L*, a*, b*) of the samples were determined using a Hunter Lab Colorimeter (MiniScan XE Plus, Hunter Associates Laboratory, Reston, VA, USA) [32]. Whey syneresis of the yoghurt samples was determined using the method described by Amatayaku et al. [33].

#### 2.4.2. Texture Analysis

Texture analysis of the yoghurt samples was carried out using a TA-XT2i texture analyser (M/s Stable Micro Systems, Goldalming, UK) fitted with a 25 kg load cell and calibrated with a 5 kg standard dead weight prior to use [34]. The yoghurt was prepared in a standard-size back extrusion container (50 mm diameter and 4 cm height) and subjected to mono-axial compression on the texture analyser, with cross head speed of 2.0 mm/s. The test mode and option used was back extrusion, with the pre-test speed as 1.0 mm s^−1^ and post-test speed as 2.0 mm s^−1^ at a temperature of 20 °C. The graphs obtained were analysed for firmness, i.e., the force for compression; stickiness, i.e., the negative peak force during withdrawal; and the work of shear and work of adhesion using the Texture Expert Exceed software (version 2.55) supplied, along with the instrument.

#### 2.4.3. Dynamic Rheological Analysis

The oscillatory rheological measurements were performed on a dynamic rheometer (MCR 52, Anton paar, Ostifildern, Germany) using cone-and-plate configuration (CP-75) of a 75 mm diameter at 20 °C. For dynamic viscoelastic determination, rheological measurements were performed using a frequency sweep test within the LVE range of the product at a constant shear rate of 1 Pa. The mechanical spectra were obtained, with us recording dynamic complex viscosity (n*), the storage modulus (*G*′), the loss modulus (*G*″) and the phase angle (tan *δ* = *G*″/*G*′) as a function of frequency (0.01 to 100 Hz) [35]. 

#### 2.4.4. Fourier-Transform Infra Red Spectroscopy

The FTIR spectra (IRAffinity-1 CE, Shimadzu, Japan) were obtained for yoghurt samples using attenuated transmission (FTIR-ATR) at 4 cm^−1^ resolutions, and the spectrum was recorded for the 400–4000 cm^−1^ range [36]. It provided information about the quantitative and qualitative analysis of chemical characteristics on the surfaces. Each spectrum consisted of an average of 60 separate scans.

#### 2.4.5. Metabolite Profiling

Metabolite profiling of the sample was carried out using gas chromatography–mass spectrometry (GC-MS) (Shimadzu GCMS TQ 8030, Kyoto, Japan) coupled with a triple quadrupole mass spectrometer (Shimadzu Corporation, Kyoto, Japan) on an Rtx 2330 Capillary column (105 m × 0.25 mm × 0.20 µm, Restek Corporation, Bellefonte, PA, USA). The samples were injected using an autosampler (AOC20i) in a split mode. The flow rate of helium gas (carrier gas) was maintained at 1.3 mL min^−1^, with the initial oven programme temperature at 100 °C and a hold time of 5 min. This temperature was then ramped up to 240 °C (rate of 4 °C min^−1^ with a holding time of 20 min). Mass spectra (*m*/*z* 50–500) were recorded at a rate of five scans per second with ionisation energy of 70 eV. The temperature of the injector and ion source was maintained at 220 °C and 230 °C, respectively. 

A freshly prepared yoghurt sample was subjected to sonication for 60 min. Around 100 µL of sonicated sample was transferred to a 1.5 mL Eppendorf tube, and 250 µL of methanol and 125 µL of chloroform were added, followed by the addition of 380 µL of chloroform and 90 µL of 0.02 M potassium chloride and centrifugation at 13,500× *g* for 10 min at 4 °C. The resulting supernatant was dried using nitrogen stream, and 50 µL of MOX reagent (Methoxamine HCl; 10 mg in 1 mL pyridoxine solution) was added. The mixture was then incubated at 30 ± 1 °C for 17 h, followed by the addition of 100 µL of the derivatizing agent MSTFA (N-methyl-N-trimethylsilyl- trifluoroacetamide) reagent. The sample was transferred to clear GC-vials, and 800 μL of hexane was added. Finally, the GC-vials were placed in the GC-MS sample holder for further analysis.

#### 2.4.6. Statistical Analysis

Data obtained from various experiments were recorded as the mean ± standard deviation (SD) and subjected to statistical analysis for us to arrive at valid and meaningful inferences. Data were analysed using one-way analysis of variance (ANOVA) followed by Tukey’s comparison test to establish the significance of differences among the mean values at a 5% level of significance, using SPSS version 20.0 software of M/s IBM Corporation. Metabolites detected were subjected to Metaboanalyst software 6.0 [37] for multivariate data analysis including principal component analysis, box-and-whisker plots, a heat map and correlation analysis. Further, the relationship between metabolites and techno-functional properties was deduced using regression analysis. 

## 3. Results and Discussion

### 3.1. Influence of Varying Total Solids Level and Type of Culture on Techno-Functional Properties of Goat Milk

Milk from the indigenous goat milch breed, *Barbari* breed, was taken and adjusted to TS levels of 12% and 16%, followed by inoculation with two different cultures, i.e., commercial culture (comprising a thermophilic yoghurt culture) and NCDC-263 (comprising a mixed yoghurt culture). All yoghurt samples were analysed for proximate composition, colour values, whey syneresis, and techno-functional properties, including flow behaviour, frequency sweep, and textural attributes. Furthermore, molecular interactions were studied using FT-IR spectroscopy. 

#### 3.1.1. Influence of Varying Total Solids Level on Techno-Functional Properties of Goat Milk Inoculated with Commercial Culture

##### Physico-Chemical Characteristics

Yoghurt samples prepared from milk containing 12% and 16% TS levels and inoculated with commercial culture @ 1 mL 1000 mL^−1^ were studied for physico-chemical characteristics in detail. The results revealed that the fat (%), protein (%), and ash (%) content of 12-A and 16-A samples exhibited a significant difference (*p* < 0.05) (Table 2). The 16-A samples had a 25% higher fat and protein content (*p* < 0.05) and 21% higher ash content (*p* < 0.05) than the 12-A samples. These findings align with the trend reported by Mahdian and Tehran [38] and Hayes et al. [39], wherein an increase in the total solids content of milk resulted in a more concentrated yoghurt, resulting in higher fat, protein, and ash contents. Further, the pH value was significantly lower (*p* < 0.05) for the 16-A sample (4.68 ± 0.01), while, acidity (% lactic acid) was significantly lower (*p* < 0.05) for the 12-A sample (0.742 ± 0.003). Reports indicate that upon decreasing the pH of milk during yoghurt preparation, kappa-casein neutralizes, collapses and loses its stabilizing power, which in turn results in the aggregation of casein particles to form a gel [40].

Furthermore, changes in colour parameters demonstrated that the 16-A samples with a higher protein content had a significantly higher (*p* < 0.05) L* value (89.252 ± 0.258) as compared to the 12-A samples (87.724 ± 0.159), which could possibly be due to increase in the scattering properties of yoghurt owing to the higher protein content of the former [41]. The a* value of yoghurt samples increased significantly (*p* < 0.05) with an increase in the TS level, ranging from −2.268 ± 0.238 in the 12-A samples to −1.629 ± 0.204 in the 16-A samples. The change in this colour component might be due to the microbial growth and the generation of new compounds [42]. A similar trend was seen in the case of the b* value which exhibited an increase (*p* < 0.05) from 9.132 ± 0.47 to 11.636 ± 0.891 with the increase in the TS level. The increase in these values might be attributed to the higher fat content (%) in the 16-A (5.35 ± 0.03) than the 12-A (3.96 ± 0.01) yoghurt sample. Syneresis is an undesirable property of set yoghurt which adversely influences its consumer acceptability. Table 2 shows that whey syneresis (%) for the 12-A yoghurt samples (38.21 ± 1.16) was significantly higher (*p* < 0.05) than the 16-A samples (21.33 ± 1.81). An increase in the total solids content could bring about changes in the composition of the yoghurt including a reduced pore size in the protein matrix, leading to a more stable gel structure which enhanced stability, and aided in retaining the liquid portion of the yoghurt, thereby reducing whey separation [43]. Further, ionic gelation of the yoghurt driven by the electrostatic interactions between carboxylic group of proteins and cations also helps the protein to form aggregates which, in turn, results in a reduction in whey syneresis [44].

##### Textural Analysis

The textural properties of the yoghurt samples were described in terms of firmness, consistency, cohesiveness, and the work of cohesion (Table 2). As the probe applies pressure and compresses the sample to a predetermined depth, the force exerted increases, initiating extrusion. The maximum force experienced is an indicator of the firmness of the sample, while the area beneath the peak represents its consistency. The results indicated that there was an approximately 33% increase in the firmness value when the TS level (%) increased from 12 (55.32 ± 4.29) to 16 (83.29 ± 8.48). This increase in firmness might be attributed to the enhanced interactions owing to the higher content of protein in the 16-A sample [45]. Moreover, significant differences (*p* < 0.05) in the consistency, cohesiveness, and work of cohesion of the yoghurt samples were also observed between the 12-A and the 16-A samples. The 16-A samples had significantly higher cohesiveness, while cohesiveness and the work of cohesion were significantly lower for the 16-A samples than the 12-A samples. A similar trend was recorded by Wen et al. [46] in set-type yoghurt treated with horseradish peroxidase.

##### Rate of Acidity Development

**Figure 1:**
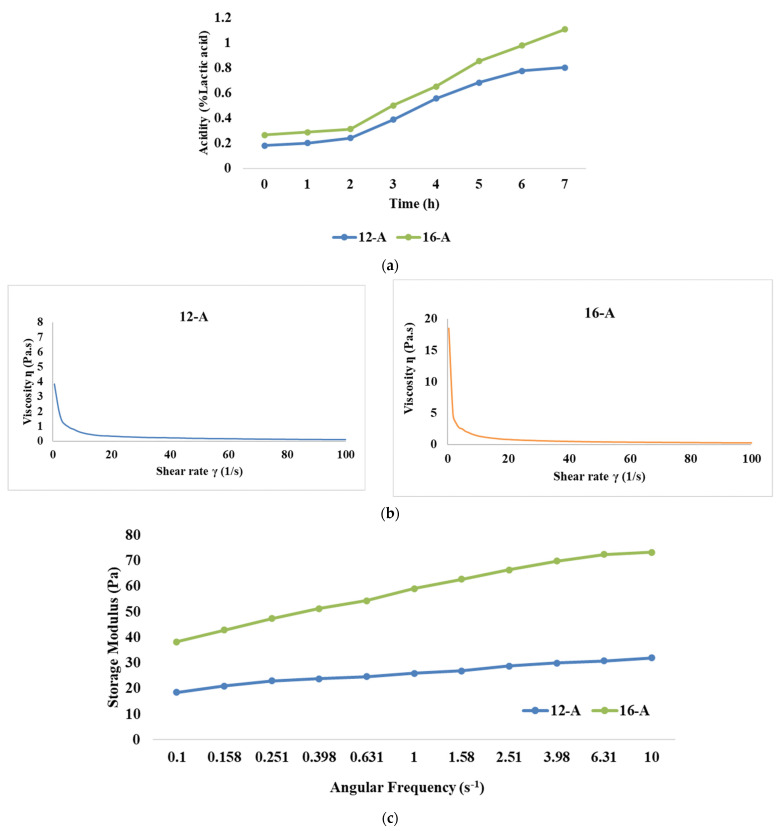

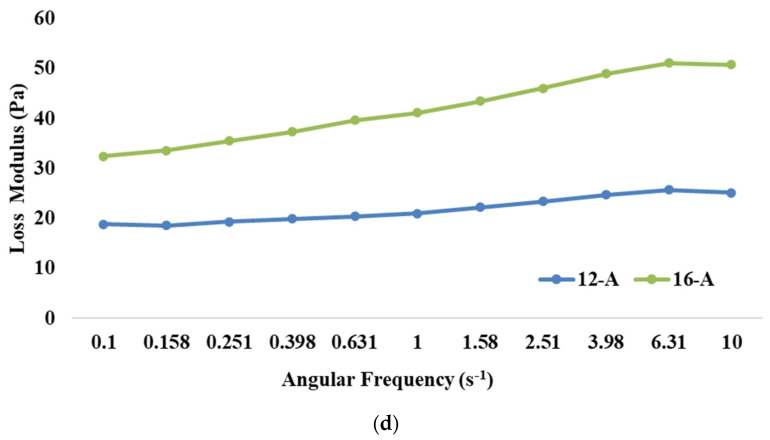
Effect of varying total solids levels on (**a**) rate of acidity development, (**b**) flow curve, (**c**) storage modulus, and (**d**) loss modulus of *Barbari* goat milk yoghurt prepared with commercial culture (12-A: milk with 12% total solids level and commercial culture; 16-A: milk with 16% total solids level and commercial culture).

Initial acidity (% lactic acid) for samples 12-A and 16-A was recorded as 0.182 and 0.266, respectively (Figure 1a. However, during the first 2 h of fermentation, the rate of acidity development (% lactic acid) was slower, with a recorded increase of 24% and 14% for the 12-A and 16-A samples, respectively. This increase could be attributed to microorganisms adapting to the substrate, and because of the growth of the yoghurt culture in the lag phase, it might have resulted in a limited increase in acidity. After the initial 2 h, the acidity increased significantly (*p* < 0.05) for both the samples due to the yoghurt culture entering the logarithmic growth phase, and the utilization of lactose at a rapid rate by lactic acid bacteria [38]. Eventually, at the end of 7 h of fermentation, the acidity levels for samples 12-A and 16-A reached a level of 0.805 and 1.108% lactic acid, with an overall increase of 77% and 74.8%, respectively. It is noteworthy that the rate of acidity development was always lower for the 16-A samples than the 12-A samples.

##### Flow Behaviour

The flow behaviour of yoghurt refers to how the viscosity or consistency of yoghurt changes in response to applied shear stress or force. The flow behaviour study of the yoghurt samples showed that the shear stress values for sample 12-A ranged between 3.85 Pa and 11.2 Pa, corresponding to viscosity values between 3.845 Pa·s and 0.112 Pa·s (Figure 1b). Similarly, the shear stress values ranged between 8.54 Pa and 22 Pa for sample 16-A and its corresponding viscosity value was between 18.485 Pa·s and 0.218 Pa·s, respectively. The median viscosity values at a shear rate of 50 s^−1^ were 0.198 Pa·s and 0.382 Pa·s for samples 12-A and 16-A, respectively. These findings corroborated the observations made by Magenis et al. [47] and Greis et al. [48], wherein yoghurt displayed shear thinning behaviour, signifying that its viscosity decreased as the shear rate increased. In general, stirred yoghurt manufactured from cow milk display higher viscosity than goat milk, as cow milk is considered to contain a lower fat content than goat milk [49]. This phenomenon is attributed to the influence of weak physical bonds, electrostatic interactions, and hydrophobic interactions within the yoghurt’s structure, along with the presence of exopolysaccharides (EPS) produced by the bacteria in the starter culture. As the shear rate increases, these bonds and interactions get disrupted, thereby leading to a decline in viscosity [50,51]. Electrostatic interactions also bring significant changes in the physico-chemical properties of protein aggregates formed due to pH decrease during yoghurt manufacturing. The unfolding and refolding of proteins due to alterations in protein sulfhydryl and disulfide bonds driven by electrostatic interactions modify the shape of protein aggregates [52] and it is reported that isoelectric solubilization/precipitation increases the gelling properties of the product [53].

##### Frequency Sweep

The frequency sweep test is employed to analyse the impact of different treatments on the structure of goat milk yoghurt. It also helps to assess whether the material exhibits frequency dependence. Our results demonstrated that the storage modulus was significantly higher (*p* < 0.05) for 16-A than 12-A (Figure 1c). Higher TS levels resulted in more solid particles in the system, which resulted in an increase in the number of intermolecular interactions and the formation of a stronger network structure, thus leading to a higher storage modulus [54]. Further, it can be observed that the higher loss modulus was also exhibited by 16-A than 12-A (Figure 1d). With a higher concentration of solids, proteins become more prominent, leading to increased energy dissipation and, consequently, a higher loss modulus [55]; however, the values of the loss modulus for both the samples were lesser than those of the storage modulus (the storage modulus value for 16-A at 10 Hz was 73.3 Pa and loss modulus was 50.7 Pa). Furthermore, changes in protein gelation, including reduced α-helix and increased β-sheets, might have also contributed to the higher loss modulus upon increasing the TS levels. This pattern indicated that yoghurt had a stronger elastic property as compared to its viscous component, thereby showing viscoelastic properties.

#### 3.1.2. Influence of Varying Levels of Total Solids and Culture on Techno-Functional Properties of Goat Milk Inoculated with NCDC 263 Culture

##### Physico-Chemical Characteristics

Yoghurt samples prepared with two different TS levels, i.e., 12% and 16% and NCDC 263 culture levels, i.e., 2% and 3%, were investigated for physico-chemical characterization in detail. Proximate composition analysis revealed that the fat, protein, and ash contents of the 12-2B, 16-2B and 12-3B, 16-3B yoghurt samples exhibited a significant difference (*p* > 0.05) at different TS levels (Table 3). Yoghurt prepared with 16% TS in milk had a significantly higher (*p* < 0.05) protein, fat and ash content. However, these variations were not statistically significant (*p* > 0.05) when the culture levels were different at the same TS level of milk. This indicates that the TS level had a significant influence (*p* < 0.05) on the proximate composition; however, the level of culture did not bring any changes (*p* < 0.05) in the proximate composition of the yoghurt samples.

Colour values for the yoghurt samples were measured in terms of the L* (lightness), a* (greenness to redness), and b* value (blueness to yellowness) and presented in Table 3. The L* value did not show significant variation (*p* > 0.05) among the yoghurt samples with different culture levels and same TS level. However, 12-3B and 16-3B had a significantly higher a* value than 12-2B and 16-2B, indicating that more culture could produce a reddish tinge in the former. Similarly, the b* value was observed with non-significant differences (*p* > 0.05) among the samples with the same TS level. Furthermore, interestingly, there were significant differences (*p* < 0.05) in the whey syneresis of all the yoghurt samples, wherein 12-2B (45.11 ± 1.09) had significantly higher (*p* < 0.05) whey separation, (%) followed by 12-3B (41.83 ± 2.42), 16-2B (27.85 ± 1.94) and 16-3B (23.34 ± 1.20). This indicates that whey syneresis was influenced by both the total solids and culture level. Further, it is noteworthy that the ionization of amino acids, including histidine, lysine, and aspartate, is strongly influenced by the pH of the product and it also affects the ionization state of free amino groups and terminal carbonyl groups, thereby causing significant changes in the protein structure and aggregation. At the iso-electric pH of proteins, low net charge occurs due to protein precipitation, which in turn is again limited due to the aggregate formation [56].

##### Textural Analysis

Firmness is considered as one of the most significant properties of set-type yoghurt as it indicates the gel strength of the yoghurt. The force required to achieve a certain deformation is measured in the form of firmness of the product. The textural attributes of the yoghurt samples demonstrated a significant variation (*p* < 0.05) in firmness, with an increase in the total solid content at same culture level (Table 2). The firmness values were significantly higher (*p* < 0.05) for 16-3B (78.32 ± 11.20) and lowest for 12-2B (45.97 ± 3.49). However, at the same TS level and different culture levels, firmness varied significantly (*p* < 0.05). A higher culture level (12-3B and 16-3B) produced more firmness in the yoghurt as compared to the 2% culture level (12-2B and 16-2B). A similar trend of an increase in the hardness of yoghurt with an increased culture level was demonstrated by Mudgil et al. [57], and they observed that a 2–2.5% culture level brought the highest firmness (*p* < 0.05) in yoghurt. It has been reported that whey proteins, α-lactalbumin and β-lactoglobulin, gains the gel strength owing to enhanced number of intermolecular disulfide bonds, which in turn directly influences the textural properties of the yoghurt. Heating results in the exposure of free thiol groups in β-lactoglobulin, which might interact with other disulfide bonds, thereby leading to the development of new disulfide bonds [58]. During texture analysis, as the probe applies pressure and compresses the sample to a predetermined depth, the force exerted increases, initiating extrusion. The maximum force experienced is an indicator of the firmness of the sample, while the area beneath the peak represents its consistency. When the probe retracts to its original position, the negative load values observed on the graph result from back extrusion, revealing information about the cohesiveness [59]. The area under the negative part of the graph is commonly referred to as the work of cohesion, representing the energy needed to disrupt the contact between the probe and the sample. Furthermore, significant differences (*p* < 0.05) were demonstrated in consistency, cohesiveness, and the work of cohesion at different total solid levels with the same culture level. A significantly higher (*p* < 0.05) consistency value was recorded for 16-3B (443.42 ± 31.66, −19.97 and −89.34), followed by 16-2B (414.04 ± 32.92). On the contrary, consistency and cohesiveness were reported with different trends and were significantly higher (*p* < 0.05) for the 12-2B samples (−8.27 ± 1.74 and −37.16 ± 5.52, respectively). These results highlight the impact of the total solids content on the texture-related properties of the yoghurt samples, showing distinct variations in all textural attributes at different TS and culture levels.

##### Rate of Acidity Development

**Figure 2:**
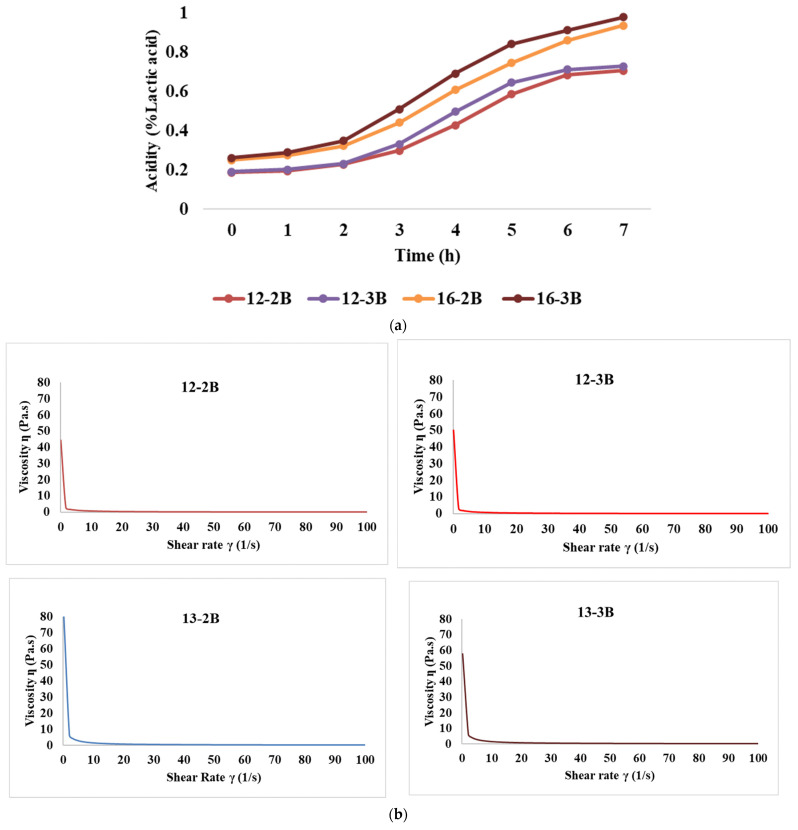

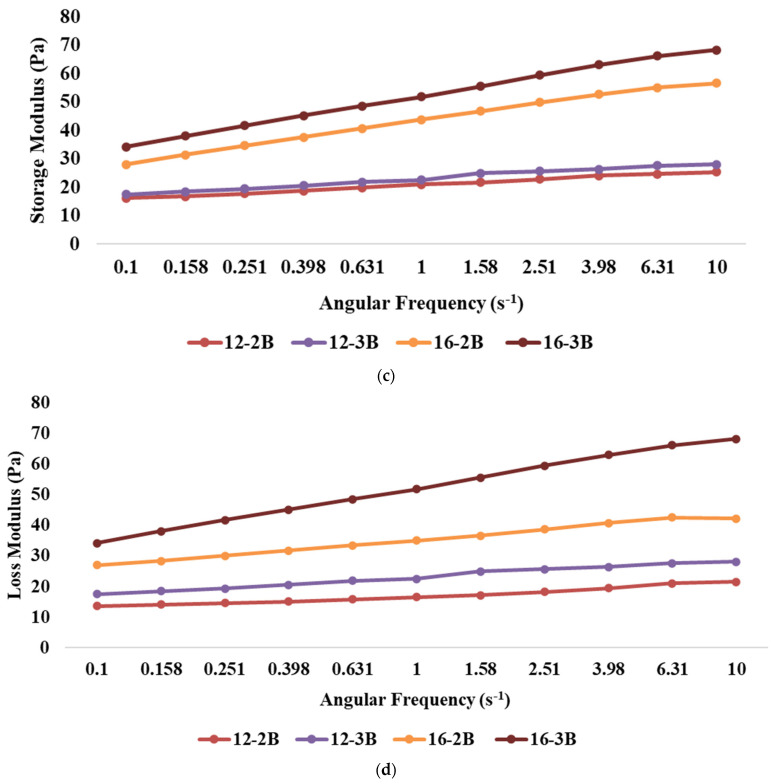
Effect of varying total solids and NCDC 263 culture levels on (**a**) rate of acidity development, (**b**) flow curve, (**c**) storage modulus, and (**d**) loss modulus of *Barbari* goat milk yoghurt (12-2B: milk with 12% total solids level and @2% NCDC-263 culture; 16-2B: milk with 16% total solids level and @2% NCDC-263 culture; 12-3B: milk with 12% total solids level and @3% NCDC-263 culture; 16-3B: milk with 16% total solids level and @3% NCDC-263 culture).

Figure 2a depicts the rate of acid development of the yoghurt samples over time. Initially, the acidity (% lactic acid) was 0.187, 0.191, 0.251, and 0.262 for the 12-2B, 12-3B, 16-2B, and 16-3B samples, respectively, which was an increase of 17.98%, 17.31%, 22.04% and 24.72%, respectively, after 2 h of fermentation. Also, after the initial 2 h, acidity increased significantly (*p* < 0.05) at both TS levels. Further, at the end of 7 h of fermentation, the acidity levels (% lactic acid) reached 0.706, 0.728, 0.937, and 0.978 for the 12-2B, 12-3B, 16-2B, and 16-3B samples, respectively. Further, 12-3B had significantly higher (*p* < 0.05) acidity as compared to 12-2B and a similar trend was observed with 16-2B and 16-3B throughout the fermentation period of 7 h. This indicates that a higher culture level induces more acid production, thereby increasing the acidity level. A similar increase in acidity levels with the increase in the culture level has been demonstrated by Yadav et al. [60] in buffalo milk set-type yoghurt.

##### Flow Curve

The flow curve graph plotted between the shear rate and shear stress (Figure 2b) depicted that shear stress values varied from 3.88 to 8.58, 4.38 to 9.32, 12.04 Pa to 16.88 Pa and 12.12 Pa to 18.54 Pa for 12-2B, 12-3B, 16-2B and 16-3B, respectively. At a shear rate of 50 s^−1^, the median viscosity values for these samples were 0.161 Pa.s, 0.180 Pa.s, 0.325 Pa·s and 0.350 Pa·s, respectively. It is noteworthy that the shear stress values and median viscosity values increased significantly (*p* < 0.05) with an increase in the TS level, while they were significantly higher (*p* < 0.05) for the samples with the 3% NCDC culture than the 2% culture. The shear thinning behaviour of fermented foods has also been recorded by Guimares et al. [61]. Further, it indicated that the yoghurt exhibited a non-Newtonian flow behaviour, which implies that the viscosity of the yoghurt was not constant but varied with the shear rate or stress applied [35]. Similar results have been reported by Ragab et al. [49], wherein all stirred yoghurt samples exhibited non-Newtonian behaviour. Increased shear rate behaviour also brings the de-cross-linking of macromolecular chains, resulting in a disrupted structure, which shows the possibility of pseudoplastic behaviour of the fermented milk gels [62].

##### Frequency Sweep

The presence of frequency dependence is indicated by a significant change in the dynamic moduli (*G*′ or *G*″) of the material with variations in frequency. The results revealed that the highest storage modulus (*p* < 0.05) was exhibited by 16-3B, followed by 16-2B, 12-3B and 12-2B (Figure 2c), indicating that an increase in the TS level resulted in a higher storage modulus, which might be attributed to the presence of more solid particles in the system. Higher TS levels also lead to increased intermolecular interactions and the formation of a stronger network structure, thus resulting in a higher storage modulus [48]. Furthermore, stronger metabolic activity in 16-3B due to a larger number of lactic acid bacteria might have contributed to a greater casein micelle-binding property, thereby resulting in a reduced pH, lesser whey syneresis and higher viscosity [63]. Similar to the storage modulus, the highest loss modulus (*p* < 0.05) was shown by 16-3B, followed by 16-2B, 12-3B and 12-2B (Figure 2d). However, the values of the loss modulus were always lower as compared to the storage modulus for all the yoghurt samples.

### 3.2. FT-IR Spectra of Yoghurt

**Figure 3:**
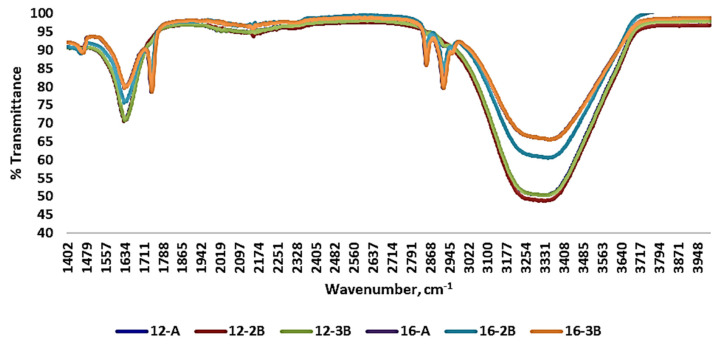
Fourier-transform infrared spectra of *Barbari* goat milk yoghurt prepared with different total solids levels of milk, types of culture and culture levels. (12-A: milk with 12% total solids level and commercial culture; 16-A: milk with 16% total solids level and commercial culture; 12-2B: milk with 12% total solids level and @2% NCDC-263 culture; 16-2B: milk with 16% total solids level and @2% NCDC-263 culture; 12-3B: milk with 12% total solids level and @3% NCDC-263 culture; 16-3B: milk with 16% total solids level and @3% NCDC-263 culture).

Various transmittance peaks were observed upon the evaluation of the FT-IR spectra of the yoghurt samples (Figure 3). It was found that yoghurt samples prepared with commercial culture (12-A and 16-A) had most of their peaks similar to those of the yoghurt prepared with NCDC 263. Functional groups were ruled out in relation to the wavenumber and associated bonds, based on the literature. Complex basic vibrations of chemical bonds are mainly responsible for bands and/or peaks at different wavenumbers [64]. Differences in the peak at a wavenumber ranging from 1550 to 1650 cm^−1^ was observed among the yoghurt samples with NCDC 263 and it reflected the amide (C=O, C-N and N-H) and water (O-H) band. Further, C=O stretching of the carbonyl group of fatty acids was also observed at the wavenumber 1750 cm^−1^. The strong peak at a wavenumber ranging from 3100 cm^−1^ to 3450 cm^−1^ indicated symmetrical stretching of the hydroxyl group (-OH) and asymmetrical stretching of the methyl group (-CH_2_), demonstrating the presence of water and the formation of sugar and fat compounds. The absorption pattern of the yoghurt samples was in concordance with Papadopoulou et al. [65] and Leal et al. [66], who studied the shelf life of yoghurt supplemented with probiotics and assessed yoghurt structure digitally, respectively.

### 3.3. Metabolite Profiling of Yoghurt and Establishing the Relationship between Metabolome and Techno-Functional Properties of Yoghurt

Based on techno-functional properties, mainly firmness, yoghurt samples with the highest (16-3B) and lowest firmness (12-2B) along with 16-A were selected for further analysis. *Barbari* milk was adjusted to 12% and 16% TS level using goat milk powder. Further, 12% TS-containing milk was inoculated with NCDC 263 @ 2% (12-2B) and 16% TS milk was inoculated with commercial culture @ 1 mL 1000 mL^−1^ (16-A) and with NCDC 263 @ 3% (16-3B). The yoghurt samples were subjected to detailed metabolite profiling and we deduced the relationship between metabolites and techno-functional properties (mainly firmness).

#### 3.3.1. Metabolite Profiling of Yoghurt Prepared from *Barbari* Milk Containing Different Total Solids Levels and Types and Levels of Starter Culture

The metabolite profiling of yoghurt samples revealed that 16-A exhibited a total of 102 metabolites. Among these, 67 metabolites were observed as identified metabolites, including sugars and related compounds (such as lactose, arabinose, allopyranose, and mannopyranose), organic acids (phenylpyruvic acid, fumaric acid, mandelic acid, and β-D-glucopyranoside), fatty acids and derivatives (stearic acid, butanedioic acid, palmitic acid, and myristic acid), while the 16-3B sample demonstrated the presence of a total of 113 metabolites and among these, 71 metabolites were characterized and quantified. The identified metabolites in the 16-3B sample spanned over different super-classes of compounds, including organic compounds (lactic acid, oxalic acid, glyceryl-glycoside, mandelic acid, and phosphates), carbohydrates (2-alpha-mannobiose, cellobiose, and β-D-lactose), and fatty acids and their derivatives (myristic acid, propenoic acid, and butanedioic acid). Further, a total of 132 metabolites were observed in the 12-2B sample and out of this, 95 metabolites were identified and associated with different classes of compounds. The metabolites in the 12-2B samples consisted of a mixture of different compounds such as sugar alcohol and its derivatives (anhydrohexitol, sorbitol, myo- insoitol, and hexaethylene glycol), organic compounds (β-D-glucopyranoside, plamitoyl glycerol, and phenyl puyruvic acid), and sugars and their derivatives (allopyranose, mannobiose, gentobiose, cellobiose, and talofuranose). A few studies have reported the presence of 56 identified metabolites in sheep yoghurt and goat yoghurt [20], while 129 metabolites were identified in goat yoghurt during the storage period of 28 days [67].

Out of the identified metabolites in each of the samples, 15 metabolites, namely, 1,5-Anhydrohexitol, Butanedioic acid, D-Lactose, Methylamine, Myo-Inositol, o-, Xylene, Palmitic Acid, Stearic acid, 10-Undecynoic acid, 2-Propenoic acid, alpha-Mannobiose, 2-Hydroxyethyl palmitate, 1-Monopalmitin, β-D-Galactofuranose and Glycerol, were differentially regulated (*p* < 0.05) in the yoghurt samples (Table 4).

#### 3.3.2. Multivariate Statistical Analysis of Differentially Expressed Metabolites in *Barbari* Yoghurt Samples

##### Principal Component Analysis and Heat Map

**Figure 4:**
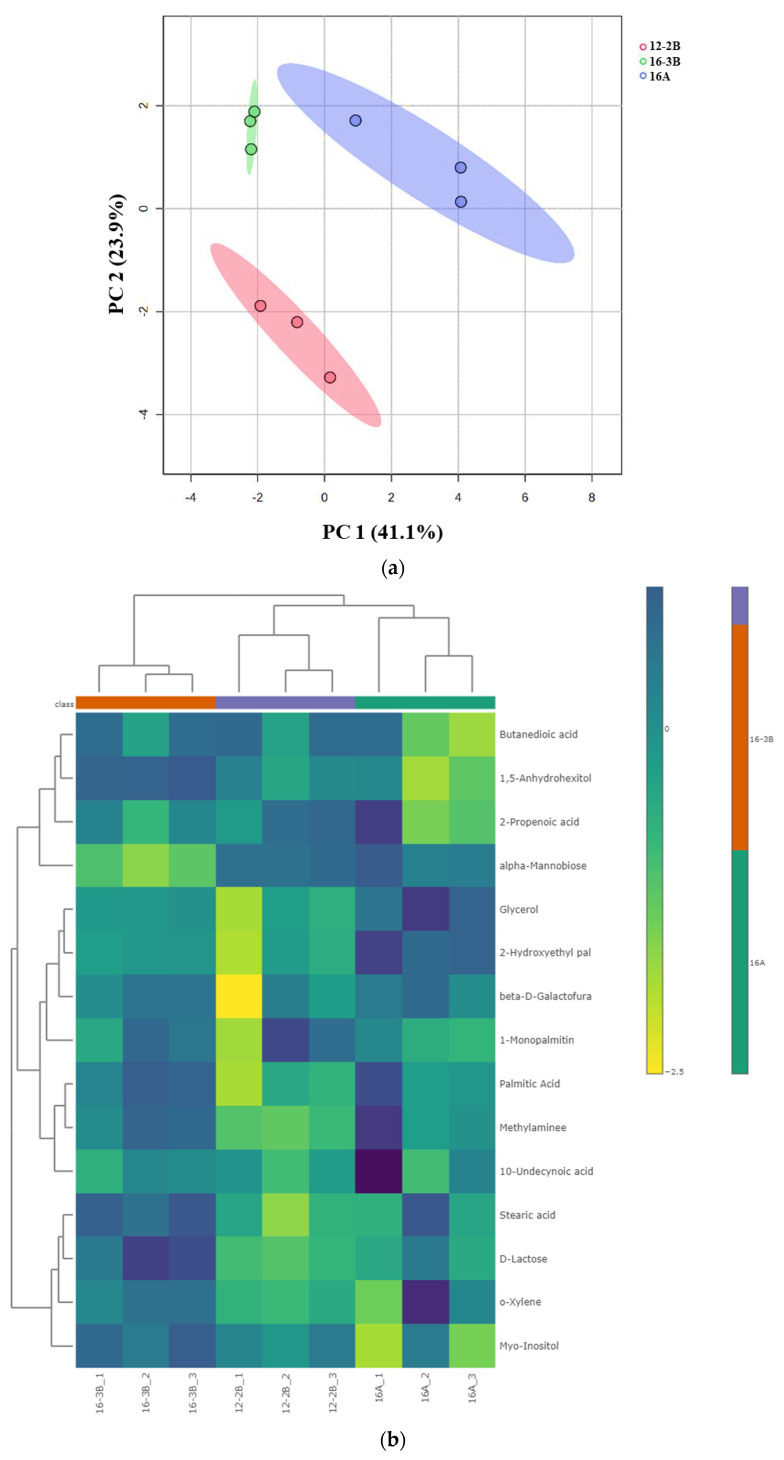
(**a**) Principal component analysis (PCA) depicting the score plot for *Barbari* goat milk yoghurt. Component 1 accounted for 41.1% of the variation in the yoghurt, which arose due to the influence of the total solids level, type of culture and culture levels; Component 2 accounted for 23.9% of the variation in the goat milk yoghurt, which arose due to the influence of the total solid level, type of culture and culture levels (16-A: milk with 16% total solids level and commercial culture; 12-2B: milk with 12% total solids level and @2% NCDC-263 culture; 16-3B: milk with 16% total solids level and @3% NCDC-263 culture). (**b**) Heat map of hierarchal clustering analysis of differential metabolites (*p* < 0.05) for *Barbari* goat milk yoghurt under the influence of different total solids, the type of culture and culture level. Coloured cells correspond to the concentration value (samples in column and compounds in row). Data presented were normalized and subjected to *T*-test/ANOVA and features were standardized to autoscaling. (16-A: milk with 16% total solids level and commercial culture; 12-2B: milk with 12% total solids level and @2% NCDC-263 culture; 16-3B: milk with 16% total solids level and @3% NCDC-263 culture).

Principal component analysis (PCA) demonstrated the variations and alterations in the metabolites under the influence of different total solids, types of culture and levels of the culture. A clear separation among the various groups of yoghurt samples was observed, indicating that the TS level in milk (12% and 16%) along with the type (NCDC 263 and commercial culture) and level of the culture (2% and 3%) had a significant effect (*p* < 0.05) (Figure 4a). The PCA plot revealed two main principal components (PCs) which explained the variation percentages of the respective groups. It was observed that PC1 explained 41.1% of the total variations, while PC2 explained 23.9% of the total variations. Using hierarchical cluster analysis, metabolites with similar properties could be grouped together and differences across metabolite groups could be identified. The goat milk yoghurt’s 15 differential metabolites (*p* < 0.05) were grouped using a Euclidean distance matrix of quantitative values in heat maps (Figure 4b). A higher concentration of metabolite in a particular sample is represented by the bright colour.

##### Correlation among Differentially Expressed Metabolites in *Barbari* Yoghurt Samples

Correlation among 15 differentially expressed metabolites in the yoghurt samples was devised to better understand the regulations of metabolites in relation to each other (Table 5). It was found that carbohydrates and other intermediates present in milk (lactose and alpha-mannobiose) were being utilized by starter culture as source of energy, and various amino-acids, amines, and peptides (methylamines) were generated during fermentation, which were then metabolized to synthesis various fatty acids and derivatives (stearic acid, palmitic acid, 10-undecyonic acid). This indicates that the upregulation of fatty acids and derivatives in yoghurt samples at one point might suggest the downregulation of a few of the carbohydrates in the sample. The results of the correlation heatmap clearly indicates that type of culture definitely influences the regulation of metabolites; however, with the trend of the alteration of metabolites, it is complex to understand the interactions among differential metabolites.

Yoghurt employs starter culture comprising of *Lactobacillus delbrueckii* subsp. *bulgaricus* and *Streptococcus thermophilus.* Both of these strains are believed to cooperate with each other to generate a number of metabolites, which are consumed by these strains to produce desirable changes in milk for its conversion to yoghurt. The most remarkable changes are in the lactose component of milk, wherein it is converted to lactose through a sequence of events taking place inside and outside the cellular system. After the transportation of lactose inside the cells, it is broken down to glucose and galactose followed by the excretion of the latter out of the cell, and the former is engaged in the glycolysis pathway [68]. Here, *Streptococcus thermophilus* takes up the galactose and produces lactic acid and carbon dioxide via the Leloir pathway. However, *Lactobacillus* lacks the genes responsible for galactose metabolism and can use only lactose and glucose. Thus, glucose is one of the metabolites which is shared by both the bacteria species. Further, glycolysis leads to the production of pyruvic acid, which in turn results in the formation of acetic acid; however, citrate, which is present as organic acid in milk, can also be metabolized by bacterial species to produce acetate [69].

##### Box-and-Whisker Plots of Differentially Expressed Metabolites in *Barbari* Yoghurt Samples

**Figure 5:**
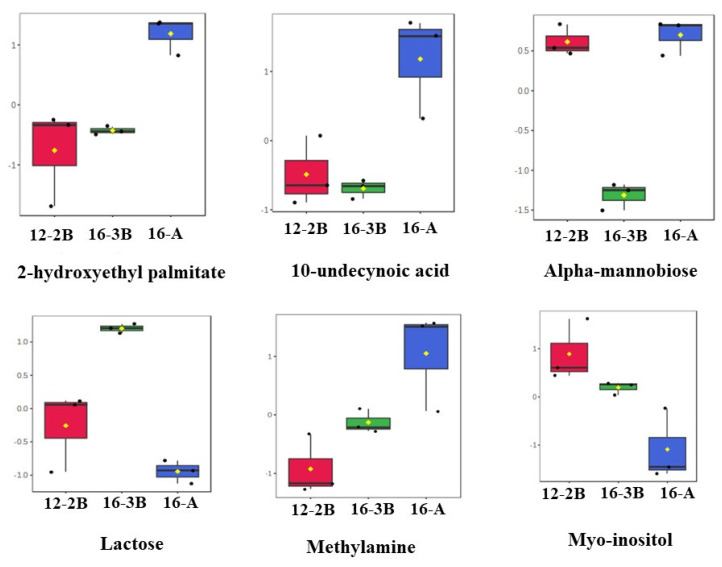
Box-and-whisker plots of differential metabolites (*p* < 0.01) present in *Barbari* goat milk yoghurt. Black dots in each plot represents the concentrations of the selected feature from all samples and mean concentration of each group is presented by yellow diamond in each box. (16-A: milk with 16% total solids level and commercial culture; 12-2B: milk with 12% total solids level and @2% NCDC-263 culture; 16-3B: milk with 16% total solids level and @3% NCDC-263 culture).

A total of 15 differentially regulated metabolites were further analysed with an ANOVA test, and it was found that out of the 15 metabolites, 6 metabolites, namely, alpha-Mannobiose, Lactose, 2-Hydroxyethyl palmitate, 10-Undecynoic acid, Myo-Inositol, and Methylamine, had a *p* < 0.01. The changes in the relative concentration of these six differentially regulated metabolites is presented in the form of box-and-whisker plots (Figure 5). The results revealed that sugars, mainly lactose and myo-inositol, were in relatively lower concentrations (*p* < 0.05) in the 16-A samples than the other two samples. Myo-inositol could be considered as one of the intermediaries during the conversion of lactose to lactic acid. These findings also suggest that a relatively lower concentration (*p* < 0.05) of sugars in the 16-A samples and a higher concentration (*p* < 0.05) of fatty acids (10-undecynoic acid and 2-hydroxyethyl palmitate) in 16-3B and 12-2B might be related to the efficient and rapid metabolism of lactose to lactic acid by the starter culture, thereby indicating the significant effect (*p* < 0.05) of the type of culture altering the metabolite profile of the yoghurt samples. Moreover, a higher concentration of fatty acids in 16-A indicates the stronger proteolytic action of the commercial culture as compared to NCDC 263 [70].

##### Pathway Impact Analysis of Differentially Expressed Metabolites in *Barbari* Yoghurt

**Figure 6:**
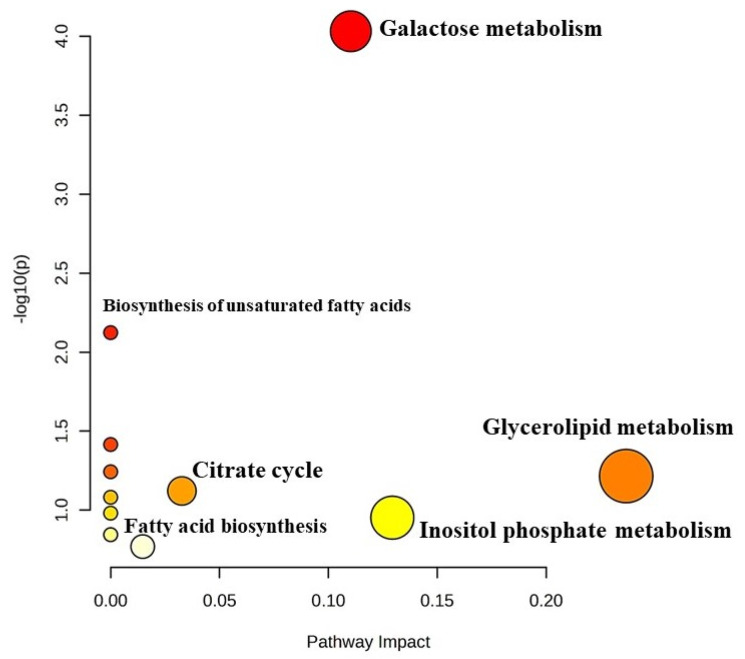
Metabolomic view map (KEGG pathway analysis) of differentially expressed metabolites in *Barbari* goat milk yoghurt. Larger size and darker colour indicate major pathway enrichment and high pathway impact values, respectively.

KEGG pathway analysis was employed to investigate the biological processes that are associated with differentially expressed metabolites (Figure 6). Pathway impact analysis revealed galactose metabolism (*p* < 0.01; 0.11 impact value), glycerolipid metabolism (*p* < 0.05; 0.2 impact value) and citrate metabolism (*p* < 0.05; 0.03 impact value). Significantly impacted galactose metabolism indicated the utilization of lactose by the starter culture to produce lactic acid, which reduced the pH of the yoghurt and brought desirable biochemical changes in the milk for the fermentation process. Metabolites, namely, lactose, myo-inositol and glycerol, were found be to be involved in galactose metabolism. Further, the metabolism of glycerolipids includes the synthesis of various glycerolipid molecules (mono-, di-, and tri-acylglycerols,), which involves glycerol as a metabolite. Hexadecanoic acid and octadecanoic acid were associated with biosynthesis of fatty acids, while succinate was involved in the citrate cycle. Therefore, the results of the analysis indicate that there are certain metabolites which regulate the pathways for the desired attributes of yoghurt.

#### 3.3.3. Relationship between Firmness and Metabolites of *Barbari* Yoghurt

In order to know the potential metabolites regulating one of the most significant techno-functional properties of yoghurt, i.e., firmness, a relationship was established between the differentially regulated metabolites (*p* < 0.01), namely, alpha-Mannobiose, Lactose, 2- Hydroxyethyl palmitate, 10-Undecynoic acid, Myo-Inositol, and Methylamine, of *Barbari* yoghurt and their respective firmness values using a regression method (Table 6). The peak area or relative concentration of each of these samples was subjected to regression analysis with the firmness value of the yoghurt sample, and it was found that methylamine (0.669) and myo-inositol (0.947) were two potential metabolites with a regression coefficient (R^2^ values) of more than 0.6. Therefore, it could be inferred that methylamine and myo-inositol could be the potential metabolites regulating the firmness of the yoghurt samples.

## 4. Conclusions

A gas chromatography-based metabolomics approach could successfully establish the relationship between the metabolome and techno-functional properties of the yoghurt.

Yoghurt samples with 16% TS exhibited the highest firmness and lowest whey syneresis than those with 12% TS. The yoghurt metabolome depicted the presence of various metabolites spanning over 4–5 major super classes. Multivariate data analysis clearly indicated the separation among metabolites of different groups, and the metabolite profiling of the yoghurt suggested that the commercial culture had more proteolytic activity than NCDC 263, as evidenced by the higher relative concentration (*p* < 0.05) of fatty acids in the samples. Among 15 differentially regulated metabolites, 2 potential metabolites, methylamine and myo-inositol, were identified which could influence the firmness of the yoghurt samples significantly. Therefore, these metabolites could be used as indicators for yoghurt with desirable textural attributes. The present study provided novel information about yoghurt metabolites, which are crucial for its firmness, whey syneresis and desired textural attributes. Overall, the present study provided fresh insights about the composition and technological attributes of yoghurt. Furthermore, studies designed to investigate the particular contribution of these metabolites would be of immense interest to dairy science.

## Figures and Tables

**Table 1:** Nomenclature of yoghurt samples.

Type of Culture	Culture Level (%)	Total Solids (%)	Sample Code
Commercial	0.1	12	12-A
		16	16-A
NCDC-263	2	12	12-2B
		16	16-2B
	3	12	12-3B
		16	16-3B

**Table 2:** Effect of varying total solids levels on proximate composition of *Barbari* goat milk yoghurt prepared using commercial culture.

S.No	Parameters		12-A ^#^	16-A ^#^
1.	Total solids (%)		12.31 ± 0.35 ^b^	16.65 ± 0.4 ^a^
2.	Fat (%)		3.96 ± 0.01 ^b^	5.35 ± 0.03 ^a^
3.	Protein (%)		2.72 ± 0.03 ^b^	3.67 ± 0.03 ^a^
4.	Ash (%)		0.72 ± 0.02 ^b^	0.91 ± 0.01 ^a^
5.	pH		4.68 ± 0.01 ^b^	4.61 ± 0.01 ^a^
6.	Acidity (% Lactic acid)		0.74 ± 0.003 ^b^	0.78 ± 0.004 ^a^
7.	Colour value	L*	87.72 ± 0.159 ^b^	89.25 ± 0.258 ^a^
8.		a*	−2.26 ± 0.238 ^b^	−1.62 ± 0.204 ^a^
9.		b*	9.13 ± 0.47 ^b^	11.63 ± 0.891 ^a^
10.	Whey syneresis (%)		38.21 ± 1.16 ^a^	21.33 ± 1.81 ^b^
11.	Back extrusion test	Firmness (g)	55.32 ± 4.29 ^b^	83.29 ± 8.48 ^a^
12.		Consistency (g·s)	338.17± 15.67 ^b^	551.51 ± 19.71 ^a^
13.		Cohesiveness (g)	−10.60 ± 1.71 ^a^	−23.80 ± 1.90 ^b^
14.		Work of cohesion (g·s)	−51.31 ± 6.73 ^a^	−94.23 ± 10.67 ^b^

^a,b^ Mean values in a row with at least one similar superscript do not differ significantly (*p* > 0.05). ^#^ 12-A: milk with 12% total solids level and commercial culture; 16-A: milk with 16% total solids level and commercial culture.

**Table 3:** Effect of varying total solids and NCDC 263 culture levels on physico-chemical characteristics and texture analysis of *Barbari* goat milk yoghurt.

S.No	Parameters		12-2B ^#^	16-2B ^#^	12-3B ^#^	16-3B ^#^
1.	Total solids (%)		12.26 ± 0.11 ^b^	16.81 ± 0.17 ^a^	12.63 ± 0.64 ^b^	16.82 ± 0.16 ^a^
2.	Fat (%)		4.00 ± 0.01 ^b^	5.33 ± 0.01 ^a^	3.99 ± 0.04 ^b^	5.31 ± 0.01 ^a^
3.	Protein (%)		2.71 ± 0.01 ^c^	3.69 ± 0.01 ^a^	2.75 ± 0.03 ^b^	3.72 ± 0.04 ^a^
4.	Ash (%)		0.71 ± 0.02 ^c^	0.92 ± 0.01 ^a^	0.72 ± 0.03 ^b^	0.93 ± 0.01 ^a^
5.	pH		4.66 ± 0.03 ^a^	4.62 ± 0.03 ^a^	4.62 ± 0.03 ^a^	4.61 ± 0.04 ^a^
6.	Acidity (% Lactic acid)		0.707 ± 0.001 ^c^	0.766 ± 0.005 ^a^	0.752 ± 0.002 ^b^	0.758 ± 0.001 ^a^
7.	Colour value	L*	87.01 ± 0.45 ^b^	88.57 ± 0.42 ^a^	87.31 ± 0.41 ^b^	88.99 ± 0.60 ^a^
8.		a*	−2.53 ± 0.10 ^b^	−1.72 ± 0.13 ^a^	−2.42 ± 0.19 ^b^	−1.71 ± 0.20 ^a^
9.		b*	9.11 ± 0.08 ^b^	11.52 ± 0.59 ^a^	9.08 ± 0.77 ^b^	11.48 ± 0.66 ^a^
10.	Whey syneresis (%)		45.11 ± 1.09 ^a^	41.83 ± 2.42 ^b^	27.85 ± 1.94 ^c^	23.34 ± 1.20 ^d^
11.	Back extrusion test	Firmness (g)	42.97 ± 3.49 ^d^	51.03 ± 4.27 ^c^	66.71 ± 9.24 ^b^	78.32 ± 7.20 ^a^
12.		Consistency (g·s)	320.13 ± 11.99 ^b^	330.85 ±11.28 ^b^	414.04 ± 32.92 ^a^	443.42 ± 31.66 ^a^
13.		Cohesiveness (g)	−8.27 ± 1.74 ^a^	−9.34 ± 1.03 ^a^	−16.05 ± 2.83 ^b^	−19.97 ± 3.58 ^b^
14.		Work of cohesion (g·s)	−37.16 ± 5.52 ^a^	−45.42 ± 10.74 ^a^	−81.01 ± 4.22 ^b^	−89.34 ± 3.99 ^b^

^a,b,c^ Mean values in a row with at least one similar superscript do not differ significantly (*p* > 0.05). ^#^ 12-2B: milk with 12% total solids level and @2% NCDC-263 culture; 16-2B: milk with 16% total solids level and @2% NCDC-263 culture; 12-3B: milk with 12% total solids level and @3% NCDC-263 culture; 16-3B: milk with 16% total solids level and @3% NCDC-263 culture.

**Table 4:** Details of differentially expressed metabolites (*p* < 0.05) in *Barbari* goat milk yoghurt.

S.No	Metabolites	Formula	Mol. Wt (g/mol)	Fold Change			VIP Score	Sub-Class	Functional Role
				16A/16-3B	16A/12-2B	16-3B/12-2B			
1.	1,5-Anhydrohexitol	C_6_H_12_O_5_	452.9	0.59258	0.69353	1.1704	0.91051	Carbohydrates and carbohydrate conjugates	Anhydro sugar
2.	Butanedioic acid	C_4_H_6_O_4_	118.09	1.096	0.84147	0.7678	0.87377	Dicarboxylic acid and derivatives	Component of citric acid or TCA cycle
3.	D-Lactose	C_12_H_22_O_11_	342.297	1.353	0.60546	3.1601	1.2004	Carbohydrates and carbohydrate conjugates	Milk sugar
4.	Methylamine	CH_3_NH_2_	31.1	1.2637	1.5831	1.2528	1.3284	Amines	Endogenous product of amine catabolism
5.	Myo-Inositol	C_6_H_12_O_6_	180.16	0.9054	0.79029	0.87287	1.2055	Alcohols and polyols	Sugar alcohol
6.	o-Xylene	C_8_H_10_	106.16	1.2936	1.481	1.1449	0.76457	Xylenes	-
7.	Palmitic Acid	C₁₆H_3_₂O₂	256.43	1.147	1.553	1.354	1.241	Fatty acids and conjugates	First acid to be produced during fatty acid synthesis
8.	Stearic acid	C_18_H_36_O_2_	284.48	1.038	1.2597	1.2136	0.99075	Fatty acids and conjugates	Hydrophobic molecule
9.	10-Undecynoic acid	C_11_H_18_O_2_	184.27	1.7359	1.4583	0.84004	0.6498	Fatty acid	Derivative of fatty acids
10.	2-Propenoic acid	C_3_H_4_O_2_	72.06	1.2727	0.88683	0.69682	0.52002	Carboxylic acid and derivatives	Derivative of fatty acids
11.	alpha-Mannobiose	C_14_H_26_O_11_	370.351	4.8394	0.94472	0.19521	0.077973	Carbohydrates and carbohydrate conjugates	Oligosaccharide
12.	2-Hydroxyethyl palmitate	C_18_H_36_O_3_	300.5	3.7207	4.506	1.2111	1.5007	Fatty acid derivative	Product of fat synthesis
13.	1-Monopalmitin	C_19_H_38_O_4_	330.5	0.95744	0.71993	0.75193	0.45036	Monoradylglycerols	Product from diacylglycerol
14.	β-D-Galactofuranos e	C_6_H_12_O_6_	180.16	1.3007	1.4337	1.1022	1.034	Carbohydrates and carbohydrate conjugates	Sugar derivative
15.	Glycerol	C_3_H_8_O_3_	92.09	2.8331	3.3826	1.194	1.5024	Carbohydrates and carbohydrate conjugates	Backbone of fatty acids

**Table 5:** Correlation table of differential metabolites (*p* < 0.05) for *Barbari* goat milk yoghurt samples.

	D- Lactose	Stearic Acid	1,5 Anhydro	Lactose	Myo-Inositol	o-Xylene	10- Undecy noic Acid	Methyl Aminee	Palmitic Acid	Beta-D-Galact Ofura	2- Hydroxy ethyl Pal	Glycerol	1-Mono Palmitin	Butane Dioic Acid	2- Propenoic Acid	Alpha-Mannobiose
D-Lactose	1	0.75168	0.07639	−0.031	−0.1213	0.48169	0.19521	0.42566	0.39513	−0.1085	0.09118	0.0538	−0.6588	−0.2321	−0.3408	−0.31037
Stearic acid	0.75168	1	0.37392	0.21962	0.22189	0.76188	−0.1022	0.13593	0.08024	−0.4366	−0.27	−0.1596	−0.8761	−0.2609	−0.5908	−0.52537
1,5- Anhydrohexitol	0.07639	0.37392	1	0.59022	0.27807	0.04084	−0.2248	0.02865	0.07439	−0.4661	−0.5267	−0.7046	−0.2768	0.06758	−0.3248	−0.5471
Lactose	−0.031	0.21962	0.59022	1	0.26084	0.27779	−0.7222	−0.3825	−0.265	0.06454	−0.4055	−0.2817	0.19369	−0.6976	−0.7986	−0.92124
Myo-Inositol	−0.1213	0.22189	0.27807	0.26084	1	0.55939	−0.7575	−0.8291	−0.8372	−0.7708	−0.9547	−0.7632	−0.0554	0.07823	−0.2629	−0.18168
o-Xylene	0.48169	0.76188	0.04084	0.27779	0.55939	1	−0.5196	−0.3995	−0.4088	−0.4446	−0.489	−0.1469	−0.5208	−0.4485	−0.6627	−0.50164
10-Undecynoic acid	0.19521	−0.1022	−0.2248	−0.7222	−0.7575	−0.5196	1	0.89438	0.81677	0.24255	0.73453	0.4455	−0.2896	0.51012	0.63099	0.59806
Methylaminee	0.42566	0.13593	0.02865	−0.3825	−0.8291	−0.3995	0.89438	1	0.96509	0.30407	0.71381	0.41499	−0.3892	0.22563	0.30752	0.18991
Palmitic Acid	0.39513	0.08024	0.07439	−0.265	−0.8372	−0.4088	0.81677	0.96509	1	0.36331	0.70406	0.39704	−0.3048	0.14912	0.29835	0.097855
beta-D- Galactofura	−0.1085	−0.4366	−0.4661	0.06454	−0.7708	−0.4446	0.24255	0.30407	0.36331	1	0.81536	0.8149	0.50043	−0.49	−0.0051	−0.012963
2-Hydroxyethyl pal	0.09118	−0.27	−0.5267	−0.4055	−0.9547	−0.489	0.73453	0.71381	0.70406	0.81536	1	0.8872	0.08705	−0.0894	0.32192	0.32654
Glycerol	0.0538	−0.1596	−0.7046	−0.2817	−0.7632	−0.1469	0.4455	0.41499	0.39704	0.8149	0.8872	1	0.09913	−0.3762	0.04984	0.17656
1- Monopalmitin	−0.6588	−0.8761	0.2768	0.19369	−0.0554	−0.5208	−0.2896	−0.3892	−0.3048	0.50043	0.08705	0.09913	1	−0.1595	0.15038	0.11225
Butanedioic acid	−0.2321	−0.2609	0.06758	−0.6976	0.07823	−0.4485	0.51012	0.22563	0.14912	−0.49	−0.0894	−0.3762	−0.1595	1	0.83644	0.77134
2-Propenoic acid	−0.3408	−0.5908	−0.3248	−0.7986	−0.269	−0.6627	0.63099	0.30752	0.29835	−0.0051	0.32192	0.04984	0.15038	0.83644	1	0.91011
alpha- Mannobiose	−0.3104	−0.5254	−0.5471	−0.9212	−0.1817	−0.5016	0.59806	0.18991	0.09786	−0.013	0.32654	0.17656	0.11225	0.77134	0.91011	1

**Table 6:** Relationship between differentially regulated metabolites with firmness values of *Barbari* goat milk yoghurt.

Metabolites	Area	Firmness (g)	R^2^
Myo-Inositol	34,86,324	69.19	0.947
Methylamine	22,42,236	69.19	0.669
alpha-Mannobiose	15,81,955	69.19	0.546
10-Undecynoic acid	62,231	69.19	0.264
2-Hydroxyethyl palmitate	4,84,350	69.19	0.166
Lactose	19,98,913	69.19	0.015

## Data Availability

The original contributions presented in the study are included in the article, further inquiries can be directed to the corresponding author.

## References

[1] Kamble K.D., Kokate P.S. (2015). Production and Keeping Quality of Yogurt from Buffalo and Cow Milk-a Traditional Milk Product of High Health Value. Indian J. Tradit. Knowl..

[2] Zhang T., Geng S., Cheng T., Mao K., Chitrakar B., Gao J., Sang Y. (2023). From the Past to the Future: Fermented Milks and Their Health Effects against Human Diseases. Food Front..

[3] Aryana K.J., Olson D.W. (2017). A 100-Year Review: Yogurt and Other Cultured Dairy Products. J. Dairy Sci..

[4] Tian H., Shi Y., Zhang Y., Yu H., Mu H., Chen C. (2019). Screening of Aroma-Producing Lactic Acid Bacteria and Their Application in Improving the Aromatic Profile of Yogurt. J. Food Biochem..

[5] Passerini D., Laroute V., Coddeville M., Le Bourgeois P., Loubière P., Ritzenthaler P., Cocaign-Bousquet M., Daveran-Mingot M.-L. (2013). New Insights into Lactococcus Lactis Diacetyl- and Acetoin-Producing Strains Isolated from Diverse Origins. Int. J. Food Microbiol..

[6] Sfakianakis P., Tzia C. (2014). Conventional and Innovative Processing of Milk for Yogurt Manufacture; Development of Texture and Flavor: A Review. Foods.

[7] Akshit F., Deshwal G.K., Sharma H., Kumar P., Maddipatla D.K., Singh M.P., Goksen G. (2024). Technological Challenges in Production of Goat Milk Products and Strategies to Overcome Them: A Review. Int. J. Food Sci. Technol..

[8] dos Santos W.M., Gomes A.C.G., Nobre M.S.d.C., Pereira M.d.S., Pereira E.V.d.S., dos Santos K.M.O., Florentino E.R., Buriti F.C.A. (2023). Goat Milk as a Natural Source of Bioactive Compounds and Strategies to Enhance the Amount of These Beneficial Components. Int. Dairy J..

[9] López-Aliaga I., Alférez M., Nestares M., Ros P., Barrionuevo M., Campos M. (2005). Goat Milk Feeding Causes an Increase in Biliary Secretion of Cholesterol and a Decrease in Plasma Cholesterol Levels in Rats. J. Dairy Sci..

[10] Nayik G.A., Jagdale Y.D., Gaikwad S.A., Devkatte A.N., Dar A.H., Dezmirean D.S., Bobis O., Ranjha M.M.A.N., Ansari M.J., Hemeg H.A. (2021). Recent Insights into Processing Approaches and Potential Health Benefits of Goat Milk and Its Products: A Review. Front. Nutr..

[11] Goswami M., Bharti S.K., Tewari A., Sharma H., Karunakara K.N. (2017). Implication of Functional Ingredients of Goat Milk to Develop Functional Foods. J. Anim. Feed. Sci. Technol..

[12] Meena P.K., Gupta V.K., Meena G.S., Raju P.N., Parmar P.T. (2015). Application of Ultrafiltration Technique for the Quality Improvement of Dahi. J. Food Sci. Technol..

[13] Sharma H., Ozogul F., Bartkiene E., Rocha J.M. (2021). Impact of Lactic Acid Bacteria and Their Metabolites on the Techno-Functional Properties and Health Benefits of Fermented Dairy Products. Crit. Rev. Food Sci. Nutr..

[14] Tamime A.Y., Robinson R.K. (2007). 5—Traditional and Recent Developments in Yoghurt Production and Related Products BT—Tamime and Robinson’s Yoghurt.

[15] German J.B., Hammock B.D., Watkins S.M. (2005). Metabolomics: Building on a Century of Biochemistry to Guide Human Health. Metabolomics.

[16] Farghal H.H., Mansour S.T., Khattab S., Zhao C., Farag M.A. (2022). A Comprehensive Insight on Modern Green analyses for Quality Control Determination and Processing Monitoring in Coffee and Cocoa Seeds. Food Chem..

[17] Tanello A.C., Silveira C.D.d.S., Carasek E., Verruck S., Prudencio E.S., Amboni R.D.M.C. (2019). Analysis of Volatile Compounds in Probiotic Yogurt during Storage through Solid-phase Microextraction Gas Chromatography. Asian J. Adv. Agric. Res..

[18] Hagi T., Kobayashi M., Nomura M. (2016). Metabolome Analysis of Milk Fermented by γ-Aminobutyric Acid–Producing Lactococcus Lactis. J. Dairy Sci..

[19] Caboni P., Murgia A., Porcu A., Manis C., Ibba I., Contu M., Scano P. (2019). A Metabolomics Comparison between Sheep’s and Goat’s Milk. Food Res. Int..

[20] Murgia A., Scano P., Cacciabue R., Dessì D., Caboni P. (2019). GC-MS Metabolomics Comparison of Yoghurts from Sheep’s and Goats’ Milk. Int. Dairy J..

[21] Allen M.M., Pike O.A., Kenealey J.D., Dunn M.L. (2021). Metabolomics of Acid Whey Derived from Greek Yogurt. J. Dairy Sci..

[22] Li D., Peng J., Kwok L.-Y., Zhang W., Sun T. (2022). Metabolomic Analysis of Streptococcus Thermophilus S10-Fermented Milk. LWT.

[23] Sun M., Yu J., Song Y., Li X., Mu G., Tuo Y. (2023). Metabolomic Analysis of Fermented Milk with *Lactobacillus delbrueckii* Subsp. Bulgaricus, *Lacticaseibacillus paracasei* Cocultured with *Kluyveromyces marxianus* during Storage. Food Biosci..

[24] Sharma H., El Rassi G.D., Lathrop A., Dobreva V.B., Belem T.S., Ramanathan R. (2021). Comparative Analysis of Metabolites in Cow and Goat Milk Yoghurt Using GC–MS Based Untargeted Metabolomics. Int. Dairy J..

[25] Mozzi F., Ortiz M.E., Bleckwedel J., De Vuyst L., Pescuma M. (2013). Metabolomics as a Tool for the Comprehensive Understanding of Fermented and Functional Foods with Lactic Acid Bacteria. Food Res. Int..

[26] Milk, Cream and Evaporated Milk—Determination of Total Solids Content (Reference Method): Bureau of Indian Standards: Free Download, Borrow, and Streaming: Internet Archive.

[27] AOAC (1984). Official Methods of Analysis.

[28] (1977). Determination of Fat by the Gerber Method, Part 2: Milk Products.

[29] (2000). AOAC Official Methods of Analysis of the Association of Official Agriculture Chemists.

[30] AOAC (1995). Official Methods of Analysis.

[31] (1961). Methods of Test for Dairy Industry (Part II) Chemical Analysis of Milk. Indian Standards Institution: Manak Bhavan.

[32] Saipriya K., Deshwal G.K., Singh A.K., Kapila S., Sharma H. (2021). Effect of Dairy Unit Operations on Immunoglobulins, Colour, Rheology and Microbiological Characteristics of Goat Milk. Int. Dairy J..

[33] Amatayakul T., Sherkat F., Shah N.P. (2006). Syneresis in Set Yogurt as Affected by EPS Starter Cultures and Levels of Solids. Int. J. Dairy Technol..

[34] Deshwal G.K., Ameta R., Sharma H., Singh A.K., Panjagari N.R., Baria B. (2020). Effect of Ultrafiltration and Fat Content on Chemical, Functional, Textural and Sensory Characteristics of Goat Milk-Based Halloumi Type Cheese. LWT.

[35] Chand P., Kumar M.D., Singh A.K., Deshwal G.K., Rao P.S., Tomar S.K., Sharma H. (2021). Low-calorie Synbiotic Yoghurt from Indigenous Probiotic Culture and Combination of Inulin and Oligofructose: Improved Sensory, Rheological, and Textural Attributes. J. Food Process. Preserv..

[36] Sharma H., Singh A.K., Rao P.S., Deshwal G.K., Singh R., Kumar M.D. (2023). A Study on Incorporation of Giloy (*Tinospora cordifolia*) for the Development of Shelf-Stable Goat Milk Based Functional Beverage. J. Food Sci. Technol..

[37] MetaboAnalyst 5.0. https://www.metaboanalyst.ca.

[38] Mahdian E., Tehrani M.M. (2007). Evaluation the Effect of Milk Total Solids on the Relationship between Growth and Activity of Starter Cultures and Quality of Concentrated Yoghurt. J. Agric. Environ. Sci.

[39] Hayes E., Wallace D., O’Donnell C., Greene D., Hennessy D., O’Shea N., Tobin J., Fenelon M. (2023). Trend Analysis and Prediction of Seasonal Changes in Milk Composition from a Pasture-Based Dairy Research Herd. J. Dairy Sci..

[40] Moitzi C., Vavrin R., Bhat S.K., Stradner A., Schurtenberger P. (2009). A New Instrument for Time-Resolved Static and Dynamic Light-Scattering Experiments in Turbid Media. J. Colloid Interface Sci..

[41] Flores-Mancha M.A., Ruíz-Gutiérrez M.G., Sánchez-Vega R., Santellano-Estrada E., Chávez-Martínez A. (2021). Effect of Encapsulated Beet Extracts (*Beta vulgaris*) Added to Yogurt on the Physicochemical Characteristics and Antioxidant Activity. Molecules.

[42] Riar H., Goel N., Singh P.K., Kumar S.S., Mishra S.K., Chawla R. (2021). Changes in Instrumental Color and Proximate Parameters in Yoghurt Fortified with Vitamin a and d Nanoemulsion during Storage. Pharma Innov. J..

[43] Vareltzis P., Adamopoulos K., Stavrakakis E., Stefanakis A., Goula A.M. (2016). Approaches to Minimise Yoghurt Syneresis in Simulated Tzatziki Sauce Preparation. Int. J. Dairy Technol..

[44] Zhou H.-X., Pang X. (2018). Electrostatic Interactions in Protein Structure, Folding, Binding, and Condensation. Chem. Rev..

[45] Kose Y.E., Altun I., Kose S. (2018). Determination of Texture Profile Analysis of Yogurt Produced by Industrial and Traditional Method. Int. J. Sci. Technol. Res..

[46] Wen Y., Kong B.-H., Zhao X.-H. (2014). Quality Indices of the Set-Yoghurt Prepared from Bovine Milk Treated with Horseradish Peroxidase. J. Food Sci. Technol..

[47] Magenis R.B., Prudêncio E.S., Amboni R.D.M.C., Júnior N.G.C., Oliveira R.V.B., Soldi V., Benedet H.D. (2006). Compositional and Physical Properties of Yogurts Manufactured from Milk and Whey Cheese Concentrated by Ultrafiltration. Int. J. Food Sci. Technol..

[48] Greis M., Sainio T., Katina K., Nolden A.A., Kinchla A.J., Seppä L., Partanen R. (2022). Physicochemical Properties and Mouthfeel in Commercial Plant-Based Yogurts. Foods.

[49] Ragab E.S., Zhang S., Korma S.A., Buniowska-Olejnik M., Nasser S.A.A., Esatbeyoglu T., Lv J., Nassar K.S. (2023). Physicochemical and Rheological Properties of Stirred Yoghurt during Storage Induced from High-Intensity Thermosonicated Goat and Cow Milk. Fermentation.

[50] Flemming H.-C. (2016). EPS—Then and Now. Microorganisms.

[51] Karimi A., Karig D., Kumar A., Ardekani A.M. (2015). Interplay of Physical Mechanisms and Biofilm Processes: Review of Microfluidic Methods. Lab a Chip.

[52] Li L., Zhao X., Xu X. (2022). Trace the Difference Driven by Unfolding-Refolding Pathway of Myofibrillar Protein: Emphasizing the Changes on Structural and Emulsion Properties. Food Chem..

[53] Zhao C.J., Gänzle M.G. (2016). Synthesis of Taste-Active γ-Glutamyl Dipeptides during Sourdough Fermentation by *Lactobacillus reuteri*. J. Agric. Food Chem..

[54] Froiio F., Cristiano M.C., Mancuso A., Iannone M., Paolino D. (2020). Vegetable-Milk-Based Yogurt-Like Structure: Rheological Properties Influenced by Gluten-Free Carob Seed Flour. Appl. Sci..

[55] Wu H., Xue R., Dong L., Liu T., Deng C., Zeng H., Shen X. (2009). Metabolomic Profiling of Human Urine in Hepatocellular Carcinoma Patients Using Gas Chromatography/Mass Spectrometry. Anal. Chim. Acta.

[56] Zhu Z., Bassey A.P., Cao Y., Ma Y., Huang M., Yang H. (2022). Food Protein Aggregation and Its Application. Food Res. Int..

[57] Mudgil D., Barak S., Khatkar B.S. (2017). Texture Profile Analysis of Yogurt as Influenced by Partially Hydrolyzed Guar Gum and Process Variables. J. Food Sci. Technol..

[58] McKerchar H.J., Clerens S., Dobson R.C., Dyer J.M., Maes E., Gerrard J.A. (2019). Protein-Protein Crosslinking in Food: Proteomic Characterisation Methods, Consequences and Applications. Trends Food Sci. Technol..

[59] Chandra M.V., Shamasundar B.A. (2015). Texture Profile Analysis and Functional Properties of Gelatin from the Skin of Three Species of Fresh Water Fish. Int. J. Food Prop..

[60] Yadav V., Gupta V.K., Meena G.S. (2018). Effect of Culture Levels, Ultrafiltered Retentate Addition, Total Solid Levels and Heat Treatments on Quality Improvement of Buffalo Milk Plain Set Yoghurt. J. Food Sci. Technol..

[61] Guimarães J.T., Silva E.K., Alvarenga V.O., Costa A.L.R., Cunha R.L., Sant’Anna A.S., Freitas M.Q., Meireles M.A.A., Cruz A.G. (2018). Physicochemical Changes and Microbial Inactivation after High-Intensity Ultrasound Processing of Prebiotic Whey Beverage Applying Different Ultrasonic Power Levels. Ultrason. Sonochemistry.

[62] Codină G.G., Franciuc S.G., Mironeasa S. (2016). Rheological Characteristics and Microstructure of Milk Yogurt as Influenced by Quinoa Flour Addition. J. Food Qual..

[63] Xiao R., Liu M., Tian Q., Hui M., Shi X., Hou X. (2023). Physical and Chemical Properties, Structural Characterization and Nutritional Analysis of Kefir Yoghurt. Front. Microbiol..

[64] Deshwal G.K., Singh R., Singh A.K., Kumar D., Sharma H. (2022). Comparative Characterisation of Ghee from Indian Camel Breeds Using GC-MS and FTIR Techniques. Int. J. Dairy Technol..

[65] Papadopoulou O.S., Argyri A.A., Kounani V., Tassou C.C., Chorianopoulos N. (2021). Use of Fourier Transform Infrared Spectroscopy for Monitoring the Shelf Life and Safety of Yogurts Supplemented with a Lactobacillus plantarum Strain with Probiotic Potential. Front. Microbiol..

[66] Leal M.A., Dias A.T., Porto M.L., Brun B.F., Gava A.L., Meyrelles S.S., Gil-Longo J., Campos-Toimil M., Pereira T.M., Vasquez E.C. (2018). Sildenafil (Viagra^®^) Prevents Cox-1/ TXA2 Pathway-Mediated Vascular Hypercontractility in ApoE-/- Mice. Cell. Physiol. Biochem..

[67] Sharma H., Ramanathan R. (2021). Gas Chromatography-Mass Spectrometry Based Metabolomic Approach to Investigate the Changes in Goat Milk Yoghurt during Storage. Food Res. Int..

[68] Sørensen K.I., Curic-Bawden M., Junge M.P., Janzen T., Johansen E. (2016). Enhancing the Sweetness of Yoghurt through Metabolic Remodeling of Carbohydrate Metabolism in *Streptococcus thermophilus* and *Lactobacillus delbrueckii* subsp. bulgaricus. Appl. Environ. Microbiol..

[69] Trimigno A., Lyndgaard C.B., Atladóttir G.A., Aru V., Engelsen S.B., Clemmensen L.K.H. (2020). An NMR Metabolomics Approach to Investigate Factors Affecting the Yoghurt Fermentation Process and Quality. Metabolites.

[70] Sharma H., Ramanathan R. (2023). GC–MS-Based Metabolomics Approach Reveals Metabolic Variations between Probiotics Incorporated Cow and Goat Milk Yoghurt. Int. J. Dairy Technol..

